# Association Between Stone Composition and Recurrence Rates Following Ureteroscopy: A Scoping Review

**DOI:** 10.7759/cureus.87602

**Published:** 2025-07-09

**Authors:** Althea O George, Mayowa Adefehinti, Minwook Lee, Ajibola A Adebisi, Ayodele Odedara, Raymond Omiko, Mobolaji Akinwale, Steven Ndonga, Adeyeye Olalekan, Abdulhameed Yusuf, Sahar Ali, Reginald Ononye, Daniel E Onobun

**Affiliations:** 1 Surgery/Urology, The Royal London Hospital, London, GBR; 2 Urology, Peterborough City Hospital, Peterborough, GBR; 3 Urology, The Royal London Hospital, London, GBR; 4 General Surgery, Epsom and St. Helier University Hospitals NHS Foundation Trust, London, GBR; 5 Trauma and Orthopaedics, Royal Shrewsbury Hospital, Shrewsbury, GBR; 6 Social Policy, University of Strathclyde, Glasgow, GBR; 7 Urology, Surgery Interest Group of Africa, Lagos, NGA; 8 Emergency Medicine, Glan Clwyd Hospital, Bodelwyddan, GBR; 9 Urological Surgery, Stepping Hill Hospital, Stockport NHS Foundation Trust, Stockport, GBR; 10 Orthopaedics and Trauma, Sheffield Teaching Hospitals NHS Foundation Trust, Warwick, GBR

**Keywords:** stent, stone composition, ureteric stones, ureteroscopy, urolithiasis, urology

## Abstract

Urolithiasis remains a significant global health burden, with high recurrence rates following intervention. Ureteroscopy is increasingly preferred due to its safety and efficacy; however, recurrence after ureteroscopy is common and may be influenced by stone composition. This scoping review aims to explore the relationship between stone composition and recurrence rates post-ureteroscopy and to identify gaps in current evidence that could inform clinical practice and research. Following the Preferred Reporting Items for Systematic Reviews and Meta-Analyses Extension for Scoping Reviews guidelines, we conducted a structured literature search (2014-2024) across PubMed, Scopus, MEDLINE, and Google Scholar. Studies were eligible if they reported on adult patients (≥18 years) undergoing ureteroscopy for renal or ureteric stones, provided stone composition data, and reported recurrence (defined radiologically, symptomatically, or via reintervention). Retrospective/prospective cohort studies, clinical trials, and case series (n >10) in English were included. Non-English, paediatric-only studies, case reports, and those lacking recurrence or composition data were excluded. In total, 13 studies met the inclusion criteria. Calcium oxalate was the most frequently reported stone type and appeared to be associated with higher recurrence rates. Reported recurrence ranged from 25.8% at 32 months to nearly 60% at 36 months, particularly in patients without metabolic follow-up. Reporting of uric acid, struvite, and cystine stones was inconsistent, limiting firm conclusions. The majority of studies were retrospective, small-scale, and lacked standardised definitions of recurrence, often conflating residual fragments with true recurrence. Language restriction and lack of granular metabolic data further limited synthesis. Stone composition appears to influence recurrence risk post-ureteroscopy, particularly for calcium-based stones. However, variability in study design, recurrence definitions, and underreporting of metabolic data reduce the strength of current evidence. Future prospective research with standardised reporting and broader linguistic inclusion is essential.

## Introduction and background

Kidney stone disease continues to place a considerable strain on urological services worldwide, not only due to its high prevalence but also because of its recurrent nature [1]. Despite advances in surgical management, long-term outcomes remain suboptimal in many patients, particularly those predisposed to recurrence. There is evidence that stone composition and metabolic profiles are determinants of recurrence risk [2]. As such, understanding its role in predicting recurrence is critical to informing postoperative follow-up and developing targeted strategies to reduce long-term stone burden [3].

The pathophysiology of stone disease is multifactorial, involving both intrinsic factors, such as genetic predisposition and metabolic derangements, and extrinsic contributors, including fluid intake, dietary habits, and environmental conditions [4]. Stone formation typically begins with urinary supersaturation of lithogenic solutes such as calcium, oxalate, and uric acid, leading to crystal nucleation, aggregation, and retention within the renal collecting system [5,6]. Clinically, patients may present with acute loin to groin colicky pain, haematuria, or chronic complications, such as recurrent urinary tract infections, hydronephrosis, or progressive renal impairment [7,8].

While multiple treatment modalities exist, including extracorporeal shockwave lithotripsy (ESWL), percutaneous nephrolithotomy (PCNL), and ureteroscopy, the latter has become a cornerstone of modern endourology due to its minimally invasive nature and high stone-free rates [9]. Advancements in ureteroscopic technologies, particularly the widespread adoption of flexible ureteroscopes and laser lithotripsy (e.g., Holmium:YAG and Thulium fibre lasers), have further enhanced procedural outcomes. Nevertheless, recurrence following ureteroscopy remains a persistent clinical challenge, often attributable to a combination of metabolic, anatomical, and behavioural factors [7,10].

Importantly, the aim of this review is not to evaluate ureteroscopy itself, but rather to use it as a clinical context in which stone composition may influence recurrence outcomes. Recurrence is affected by factors such as stone type, metabolic risk profiles, obesity, diabetes, young age, and personal or family history of nephrolithiasis [10]. Despite the established role of composition in guiding preventive strategies, post-ureteroscopy follow-up remains variable and often lacks metabolic assessment or structured patient counselling [11,12]. Hence, this review aims to: (i) synthesise recurrence patterns by stone composition following ureteroscopy; (ii) evaluate how recurrence risk varies across stone types; and (iii) identify gaps to inform future research and practice.

## Review

Methodology

Overview of Methodology

This scoping review was conducted in accordance with the Preferred Reporting Items for Systematic Reviews and Meta-Analyses Extension for Scoping Reviews (PRISMA-ScR) guidelines developed by Tricco et al. and was guided by the methodological frameworks proposed by Arksey and O'Malley and further refined by the Joanna Briggs Institute (JBI) [13,14]. These frameworks were selected to ensure transparency, methodological rigour, and reproducibility in mapping existing literature on stone composition and recurrence after ureteroscopy.

Protocol and Registration

This scoping review followed the PRISMA-ScR guidelines developed by Tricco et al. [14], ensuring transparency and methodological rigour. The protocol was informed by a preliminary literature scan and refined with input from experts in urology and systematic reviews. Although the review protocol was not registered, this decision aligns with PRISMA-ScR flexibility for exploratory scoping designs. Ethical approval was not required, as only publicly available data were used. All steps, from search to synthesis, were aligned with PRISMA-ScR to ensure a transparent and reproducible process.

Eligibility Criteria

This review was structured using the Population, Concept, and Context (PCC) framework recommended by the JBI.

Population: Adult patients (aged ≥18 years) undergoing ureteroscopy for ureteric or renal stones.

Concept: Stone composition (fully or partially reported) and recurrence rates defined radiologically, symptomatically, or via reintervention.

Context: Studies published between January 2014 and April 2024 in English.

Eligible study designs included retrospective and prospective observational studies, clinical trials, and large case series (n > 10). Exclusion criteria comprised case reports, reviews, editorials, conference abstracts without full text, non-English publications, and studies not primarily focused on ureteroscopy or without data on stone composition and recurrence.

Search Strategy and Information Sources

A comprehensive literature search was conducted across the following four electronic databases: PubMed, Scopus, MEDLINE (via Ovid), and Google Scholar. The search spanned publications from January 2014 to April 2024 to reflect current endourological practices. Grey literature was identified through Google Scholar, institutional repositories, and clinical trial registries. Reference lists of included studies were also screened to enhance coverage.

The search strategy used Boolean operators and MeSH terms, combining keywords for ureteroscopy, stone composition, and recurrence. Filters were applied to limit results to human studies, English-language studies, and relevant publication types. The final search was conducted on 30 April 2024. Full search strings are detailed in Table 1 and Appendix A.

**Table 1:** Search strategy.

Search component	Keywords/Search terms	Boolean operators
Concept 1: Procedure	“ureteroscopy” OR “endoscopic stone removal”	OR
Concept 2: Stones	“urolithiasis” OR “renal calculi” OR “stone composition”	OR
Concept 3: Outcome	“recurrence” OR “recurrent stones” OR “stone recurrence”	OR
Combined search	(“ureteroscopy” AND “stone composition” AND “recurrence”)	AND

The third phase involved grey literature searches via Google Scholar, institutional repositories, urology conference abstracts, and clinical trial registries to identify unpublished or ongoing studies. Reference lists of included articles and reviews were also manually searched to minimise publication bias.

Selection of Sources of Evidence

Study selection followed a structured two-phase screening process, i.e., title/abstract review and full-text assessment, guided by established scoping review frameworks. Two independent reviewers applied predefined inclusion criteria, resolving discrepancies through discussion or third-party adjudication to ensure objectivity and reduce bias.

Data Charting Process

A total of 13 studies were included in this scoping review after screening 69 records from databases and additional sources, with duplicates removed and exclusions documented. Data were charted based on author, year, country, study design, sample size, stone type, recurrence definitions, follow-up duration, recurrence rates, and recurrence-free survival.

Data Synthesis Strategy

Given the heterogeneity in study designs, recurrence definitions, follow-up durations, and outcome reporting across the included studies, a formal meta-analysis was not feasible. Instead, a descriptive, narrative synthesis was employed to summarise patterns in stone composition and recurrence rates. This approach is consistent with the aims of scoping review methodology, which prioritises breadth of evidence mapping over statistical aggregation. While this limits the ability to draw definitive conclusions or pooled estimates, it allows for the identification of key trends, thematic patterns, and gaps in the literature. Future studies with standardised methodologies and consistent outcome measures would be required to support a meta-analytic approach and strengthen the evidence hierarchy.

In accordance with PRISMA-ScR and JBI guidelines, no formal risk of bias assessment was undertaken, as the objective of this scoping review was to characterise the breadth and nature of available evidence rather than evaluate study quality. Most included studies were retrospective, and findings should be interpreted within that context.

Synthesis of Results

A total of 69 records were identified through database searches and supplementary sources. After removing duplicates, 39 unique studies remained and were screened for eligibility. Title and abstract screening excluded 11 studies that did not meet the inclusion criteria, leaving 28 for full-text review. Following a detailed assessment, 13 studies met all inclusion criteria and were included in the final synthesis. Reasons for exclusion at the full-text stage included absence of recurrence data, lack of stone composition detail, or insufficient sample size.

Data were synthesised using a descriptive analytical approach. Studies were grouped by stone composition, recurrence definitions, and follow-up duration to identify thematic patterns and common findings across heterogeneous data sources. Evidence mapping is presented in Table 2.

**Table 2:** Evidence mapping distribution.

Reason for exclusion	Number of articles	Further explanation
Irrelevant to ureteroscopy or urolithiasis	5	These articles did not focus on ureteroscopy as a treatment modality for stone disease
No data on stone composition	3	Studies lacked detailed information about the chemical or mineral composition of stones
No outcome data on recurrence	2	Articles failed to report recurrence rates or follow-up outcomes after ureteroscopy
Small case series (n ≤ 10)	1	The study did not meet the sample size threshold defined for inclusion
Review articles, commentaries, or editorials	2	Non-empirical literature that did not present original data was excluded
Total	13	

Results

PRISMA-ScR Flow Diagram

**Figure 1:**
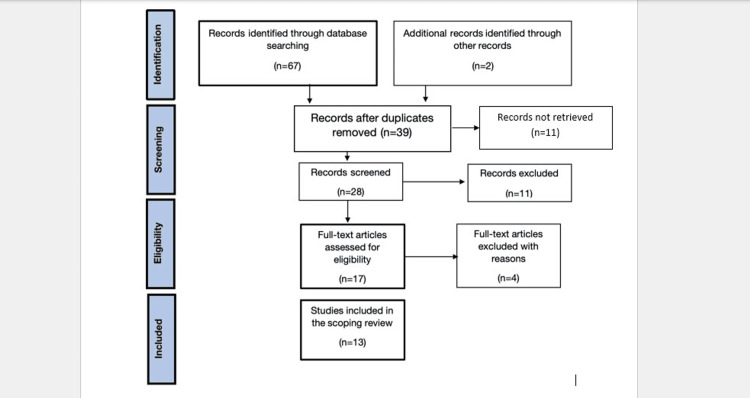
Preferred Reporting Items for Systematic Reviews and Meta-Analyses Extension for Scoping Reviews (PRISMA-ScR) flow diagram.

The PRISMA-SCR flow diagram presented in Figure 1 illustrates the detailed explanation of the search decision and the record selection process.

Summary of Study Characteristics

The included studies varied in design and setting, with most being retrospective observational studies. Geographic representation spanned Europe, Asia, North America, and Africa. Sample sizes ranged from 45 to 300 patients. Table 3 summarises key characteristics, including study location, methodology, and objectives.

**Table 3:** Summary of the studies included in the scoping review.

Year	Authors	Title	Location	Methodology	Objectives
2024	Skolarikosa et al. [2]	Metabolic evaluation and recurrence prevention for urinary stone patients: An EAU guidelines update	Ireland (UK)	Qualitative	To define patients who are at a high risk of recurrence of urolithiasis by conducting a literature review that proposes to delineate diagnostic and therapeutic algorithms for each type of stone, with the aim of and to clarify general guidelines and recommendations for the prevention of recurrence
2022	Baowaidan et al. [10]	Incidence and risk factors for urolithiasis recurrence after endourological management of kidney stones: a retrospective single-centre study	France	Qualitative	To determine the recurrence rate of lithiasis after endourological management of nephrolithiasis and identify the risk factors for these recurrences
2014	Vernez et al. [15]	Nephrolithometric scoring systems to predict outcomes of percutaneous nephrolithotomy	USA	Qualitative	To develop a user-friendly system (S.T.O.N.E. Score) to quantify and describe stone characteristics provided by axial CT scan to predict ureteroscopy outcomes and to evaluate the characteristics that are thought to affect stone-free rates
2017	Oliver et al. [16]	Successful ureteroscopy for kidney stone disease leads to resolution of urinary tract infections: prospective outcomes with a 12-month follow-up	Southampton (UK)	Qualitative	To investigate the resolution of urinary tract infection with the successful treatment of kidney stone disease
2017	Aggarwal et al. [17]	Renal stones: a clinical review	India	Qualitative	To conduct a clinical review of the prevalence of urinary tract stones in India and provide more information on the illness
2018	Alelign et al. [18]	Kidney stone disease: an update on current concepts	Ethiopia	Qualitative	To evaluate the increasing rate of urological disorders of human health, affecting about 12% of the world population. Also, to explore the relationship between stone recurrence and an increased risk of end-stage renal failure
2019	D’Costa et al. [19]	Leave no stone unturned: defining recurrence in kidney stone formers	USA	Qualitative	To explore and investigate the various manifestations of kidney stone recurrence and the reported rates of kidney stone recurrence in various clinical settings
2019	Fontenelle et al. [20]	Kidney stones: treatment and prevention	Brazil	Quantitative	To investigate and assess the treatment and prevention of kidney stones, as they are becoming a prevalent disorder, with an annual incidence of eight cases per 1,000 adults
2020	Nevo et al. [21]	Patients treated for uric acid stones reoccur more often and within a shorter interval in comparison to patients treated for calcium stones	Canada	Quantitative	To investigate the association between stone composition and recurrence rate in a well-characterized group of patients
2020	Lane et al. [22]	Correlation of operative time with outcomes of ureteroscopy and stone treatment: a systematic review of literature	Southampton (UK)	Qualitative	To present the latest evidence related to the impact of increased operative times in retrograde intrarenal surgery and identify possible important factors that can facilitate ureteroscopy procedures
2023	Kim et al. [23]	Real-world practice stone-free rates after ureteroscopy: variation and outcomes in a surgical collaborative	Michigan (USA)	Mixed-method	To evaluate the stone-free rate for ureteroscopy and its predictors across diverse surgeons in Michigan
2023	Ripa et al. [24]	Role of ureteroscopy (URS) and stone treatment in patients with recurrent UTIs: outcomes over a 10-year period	Southampton (UK)	Quantitative	To assess whether the eradication of kidney stones might result in a substantial reduction in the onset of recurrent urinary tract infections
2024	Achar et al. [25]	Indications for intervention in patients undergoing ureteroscopic therapy for ureteric calculus: a cross-sectional study	India	Quantitative	To analyse the various indications for surgery, whether MET was used or not, if used-its details, operative findings at ureteroscopy, including the reason for the failure of MET

Frequency of Stone Composition

Calcium oxalate stones, particularly the monohydrate subtype, were the most frequently reported across studies. Baowaidan et al. identified calcium oxalate monohydrate in 23.2% of patients, followed by mixed stones (20.5%), calcium oxalate dihydrate stones (6.8%), and uric acid stones (5.8%) [10]. Other studies, including Kim et al. and Skolarikos et al., corroborated the predominance of calcium oxalate stones [2,23]. Uric acid, struvite, cystine, and brushite stones were underrepresented or not stratified in several studies, highlighting a limitation in composition-specific analysis [23,25].

Recurrence Stratified by Stone Type

Recurrence rates varied widely across the studies. For example, Baowaidan et al. reported a 25.8% recurrence rate over a median of 32 months, while Skolarikos et al. cited recurrence approaching 60% at 36 months in patients without metabolic follow-up [2,10,23]. Calcium oxalate stones were consistently associated with higher recurrence risk, though few studies stratified recurrence rates by specific subtypes [23].

Nevo et al. found that uric acid stone-formers experienced recurrence earlier and more frequently than those with calcium oxalate stones [21]. However, most studies lacked sufficient detail on recurrence rates for non-calcium stone types, limiting generalisability. This underreporting underscores the need for standardised compositional reporting.

Reporting and Follow-Up Gaps

A key limitation across included studies was heterogeneity in recurrence definitions and follow-up duration. Follow-up periods ranged from four weeks to over five years, and recurrence was variably defined as radiological detection, symptomatic presentation, or need for re-intervention. Early recurrences (<6 weeks) were often not differentiated from residual fragments, which may have inflated reported recurrence rates.

Furthermore, metabolic evaluation was inconsistently reported. Only a minority of studies incorporated metabolic profiling or preventive strategies. This gap highlights the limited integration of recurrence prevention protocols in routine follow-up after ureteroscopy.

Overall, the review identifies recurring methodological limitations: inconsistent recurrence definitions, underreporting of non-calcium stone types, and inadequate follow-up strategies. Addressing these issues is critical for improving recurrence risk stratification and personalising postoperative surveillance.

Discussion

Interpretation of Findings

This scoping review highlights the prevailing patterns of stone composition and their potential association with recurrence following ureteroscopy, while also revealing substantial gaps in consistency and methodology across studies. Notably, calcium-based stones, especially calcium oxalate, were consistently identified as the dominant stone type, aligning with global epidemiological data suggesting they account for over 70% of urolithiasis cases. Their high recurrence risk, as demonstrated by Skolarikos et al. and Baowaidan et al., reinforces the importance of long-term surveillance and metabolic evaluation, particularly in patients forming calcium oxalate monohydrate stones [2,10].

This association underscores a key theme: recurrence is not solely the result of surgical failure or residual fragments, but rather a reflection of ongoing metabolic imbalance [7,26]. Therefore, managing stone disease extends beyond the procedural endpoint; it necessitates a multidisciplinary approach involving urologists, nephrologists, dieticians, and primary care providers. Yet, despite the established link between stone composition and metabolic profile, only a minority of studies integrated metabolic workup or preventive strategies such as dietary counselling or pharmacotherapy into their analysis.

Beyond calcium oxalate, the representation of other stone types, such as uric acid, struvite, cystine, and mixed stones, was inconsistent and often inadequately reported. Uric acid stones, though potentially responsive to alkalinisation therapy, were rarely stratified in recurrence outcomes [21]. Similarly, infection-related struvite stones and hereditary cystine stones were too infrequent to draw meaningful conclusions.

Another major limitation across the reviewed literature was the lack of standardisation in defining and measuring recurrence. Follow-up durations varied from as short as four weeks to several years, and recurrence definitions ranged from imaging-confirmed new stones to symptomatic presentation or treatment failure. Notably, recurrence identified within very short intervals, such as four weeks postoperatively, likely represents residual fragments rather than true recurrence. For instance, Baowaidan et al. reported a 25.8% recurrence over a median of 32 months, while Skolarikos et al. cited rates nearing 60% over three years in patients without metabolic correction [2,10]. Without consistent assessment of stone-free status post-ureteroscopy, especially in the early postoperative period, these findings risk misclassification, which undermines comparability and weakens generalisability across populations.

Ultimately, while this review affirms that calcium oxalate stones are both the most prevalent and most prone to recurrence following ureteroscopy [3], the strength of this conclusion is limited by inconsistencies in study design, outcome reporting, and lack of integration between stone composition and broader metabolic context. Future research must aim to stratify recurrence risk by stone type using standardised definitions and incorporate comprehensive postoperative metabolic assessment. Doing so will allow for more precise follow-up strategies and better-informed recurrence prevention protocols in clinical urological practice.

Clinical Implications

The findings of this scoping review carry important implications for the clinical management of patients undergoing ureteroscopy. While ureteroscopy remains an effective intervention for stone clearance, the evidence highlights that recurrence risk, particularly in calcium oxalate stone-formers, must be anticipated and managed beyond the operative episode. Stone composition is not merely a descriptive pathology but a clinical marker that should guide long-term surveillance, preventive strategies, and patient education [27].

The consistently high recurrence observed in patients with calcium oxalate stones, as reported in some studies, suggests the need for structured, stone-type-specific follow-up. These patients often have underlying metabolic derangements, such as hyperoxaluria or hypocitraturia, which are modifiable but frequently unassessed. Therefore, integrating routine stone analysis, metabolic evaluation, dietary counselling, and pharmacological prophylaxis, such as thiazides or citrate therapy, into the postoperative care plan is essential to reduce recurrence risk [2,28].

However, the review also exposes a recurring clinical oversight: inconsistent or absent stone composition reporting. In many studies, stone fragments were either not retrieved or not analysed, representing a missed opportunity to personalise care. This gap is particularly concerning in high-risk populations, where early identification of stone type could directly inform preventive strategies [29].

Equally important is the role of patient education. Post-ureteroscopy counselling remains sporadic, yet structured education on fluid intake, dietary modification, and medication adherence is proven to reduce recurrence. Preventing future stone episodes requires more than surgical skill; it demands active, multidisciplinary collaboration involving urologists, nephrologists, dietitians, and primary care providers [30,31].

Finally, these clinical insights highlight a research gap [4,31]. There is a pressing need for prospective studies and randomised controlled trials evaluating the impact of stone composition-based surveillance on recurrence rates and long-term outcomes. Much of the current evidence is retrospective and lacks granularity on follow-up practices and intervention efficacy. Moving forward, routine integration of stone analysis and metabolic profiling should be standard care in endourology to shift the paradigm from stone removal to recurrence prevention [19,32].

Research Gaps and Future Directions

This review revealed key gaps limiting the strength of current evidence on stone composition and recurrence after ureteroscopy. Inconsistent or incomplete reporting of stone composition was common, hindering accurate risk stratification. Definitions of recurrence varied widely, radiological, symptomatic, or intervention-based, making cross-study comparisons difficult. Follow-up durations lacked standardisation and rarely addressed late or side-specific recurrence. Most studies were retrospective and lacked integration of patient-specific factors such as metabolic profiles and comorbidities, limiting insights into personalised recurrence risk and long-term outcomes. Prospective, standardised research is needed to address these limitations.

Strengths and limitations

This scoping review employed a comprehensive, multi-database search strategy and adhered to PRISMA-ScR guidelines, enhancing its methodological transparency and reproducibility. By focusing specifically on stone composition and recurrence after ureteroscopy, it addresses an important but underexplored clinical issue. The inclusion of studies across diverse populations adds to the generalisability of findings, and the synthesis of recurrence data by stone type offers practical insights.

However, several limitations must be acknowledged. First, the heterogeneity in study design, follow-up duration, recurrence definitions, and outcome reporting limits the comparability and strength of conclusions. Most included studies were retrospective and lacked uniform definitions of recurrence, making synthesis challenging. Second, the absence of consistent stone-free rate reporting is a significant limitation. Without clear documentation of post-procedural stone-free rate, particularly in studies with short follow-up durations, it is difficult to differentiate between true recurrence and residual fragments, potentially confounding recurrence estimates.

Additionally, patient-level factors such as metabolic evaluations, comorbidities, or adherence to preventive strategies were infrequently reported. Language bias is also present, as only English-language studies were included due to translation constraints. Despite these limitations, the review provides a valuable foundation for informing tailored follow-up protocols and future research design.

## Conclusions

Calcium oxalate stones, particularly the monohydrate subtype, were the most frequently reported in the literature and appeared to be associated with higher recurrence following ureteroscopy. However, recurrence data were inconsistently stratified by stone composition, and most evidence was drawn from retrospective studies. Other stone types, such as uric acid, calcium phosphate, cystine, and struvite, were underreported or lacked sufficient recurrence data to allow definitive conclusions. These findings highlight the need for stone-type-informed follow-up strategies, with calcium oxalate stone-formers potentially benefiting from more structured metabolic evaluation, dietary counselling, and imaging surveillance. However, due to limited and heterogeneous data for non-calcium stones, recommendations for these subtypes remain tentative. This review underscores the importance of routine stone analysis and calls for more prospective research using standardised definitions of recurrence and consistent stone composition reporting. Such efforts are essential to advance personalised post-ureteroscopy care and improve long-term outcomes in patients with urolithiasis.

## Appendices

Appendix A

**Table 4:** Full search strategy.

Search component	Keywords/Search terms	Boolean operators/Notes
Concept 1: Procedure	“ureteroscopy” OR “endoscopic stone removal” OR “URS”	OR
Concept 2: Stones	“urolithiasis” OR “renal calculi” OR “kidney stones” OR “stone composition”	OR
Concept 3: Outcome	“recurrence” OR “stone recurrence” OR “recurrent stones” OR “relapse”	OR
Combined Boolean search string	(“ureteroscopy” OR “URS”) AND (“urolithiasis” OR “renal calculi” OR “stone composition”) AND (“recurrence” OR “recurrent stones”)	AND
Filters applied	Language: English publication; Date: January 2014 to April 2024; Population: humans; Study Type: clinical trials, observational studies, large case series (>10 patients)	AND
Databases searched	PubMed, Scopus, MEDLINE (via Ovid), Google Scholar	AND
Date of last search	April 24, 2024	-

Appendix B 

**Table 5:** Data charting process.

Author(s), year, country	Study design	Sample size	Stone types and proportions	Recurrence definitions and follow-up duration	Recurrence rates per stone type	Recurrence-free survival
Skolarikosa et al. 2015, Ireland [2]	Systematic literature review (qualitative)	European Association of Urology (EAU) urolithiasis guidelines, covering the timeframe between 1976 and June 2023	Calcium oxalate stones, with a high risk of recurrence and complications	The recurrence definition was considered from evidence that patients who adhere to targeted medical treatment seem to experience less stone growth or regrowth of residual fragments, and may be discharged after 36–48 months of follow-up on disease on imaging	Findings indicated that about 40% of patients with metabolic abnormalities not on medication remained stone-free after 3 years of follow-up; more extensive follow-up is recommended	To ensure recurrence-free survival, the findings suggest that for every patient with urolithiasis, an attempt to analyse the stone and general instructions on preventing recurrence are recommended
Baowaidan et al. 2022, France [10]	Retrospective cohort study (qualitative)	Patients who were treated for nephrolithiasis by endourological management from May 2014 to January 2017 in the Department of Urology, Angers University Hospital	The most common types of stones were calcium oxalate monohydrate stones (n = 44, 23.2%), mixed stones (n = 39, 20.5%), including mixed calcium oxalate (n = 10; 8.5%), calcium oxalate dihydrate stones (n = 13, 6.8%) and uric acid stones (n = 11, 5.8%)	At the end of a median follow-up of 32 months (range, 13–61 months), 49 patients (25.8%) had a recurrent stone. The follow-up in the univariate analysis and the risk factors for recurrence were BMI greater than 25 kg/m^2^ (HR = 2; p < 0.05), diabetes (HR = 3.73; p < 0.008), and smoking (HR = 3.1; p < 0.039). However, age (HR = 0.96; p < 0.003) and high blood pressure (HR = 0.37; p < 0.027) were protective factors.	In multivariate analysis, diabetes, smoking, hypertension, and age are still risk factors for recurrence	In this study, 25.8% of patients had recurrent stone disease after endourological management with a median follow-up of 32 months. The study findings showed that diabetes and smoking were risk factors for recurrence, while age and blood hypertension are protective factors that decreased the risk of recurrence
Vernez et al. 2016, USA [15]	The S.T.O.N.E. model (S) ize, (T)opography (location of stone), (O)bstruction, (N)umber of stones present, and (E)valuation of Hounsfield Units (Qualitative)	The S.T.O.N.E. Score was applied to 200 rigid and flexible ureteroscopies performed at The Never Health Medical Centre	The logistic model found was SFR = 1/(1+e^(-z)), where z = 7.02-0.57*Score with an area under the curve of 0.764. A S.T.O.N.E. Score ≤9 points obtains stone free rates >90% and typically falls off by 10% per point thereafter	The S.T.O.N.E. Score is a novel assessment tool to predict the stone-free rate in patients who require ureteroscopy for the surgical therapy of ureteral and renal stone disease	The features of S.T.O.N.E. are relevant in predicting stone-free rates with ureteroscopy. Size, location, and degree of hydronephrosis were statistically significant factors in multivariate analysis	Stone location is an important factor in the success of ureteroscopy, especially lower pole stones (5, 6, 18). Although the visualisation of these stones may be possible with flexible endoscopes, the acute pelvic infundibular angle may prevent access to the stone
Oliver et al. 2017, UK [16]	Prospective cohort study (Qualitative)	Between March 2012 and July 2016, consecutive patients who underwent ureteroscopy for stone disease with a history of recurrent urinary tract infections or culture-proven urinary tract infections	The mean cumulative stone size was 16 mm (range = 3–107 mm), and a positive preoperative urine culture was found in 81 (79%) patients.	While the overall SFR was 96%, the total complication rate was 12.6% (n = 13), and these were all Clavien I/II complications. At follow-up, the stone-free rate and infection-free rate were 96% and 88% at three months and 82% and 71% at 12 months, respectively (p < 0.001)	While almost three-quarters of patients were stone and infection free at 12 months, the majority of those with stone recurrence also had recurrence of their urinary tract infection	The majority of patients will remain infection-free at the 12-month follow-up if they are stone-free after their initial treatment
Aggarwal et al. 2017, India [17]	Expository cohort study (qualitative)	Persons aged 20–49 years	The most common being the calcium oxalate stones. Various dietary, non-dietary, and urinary risk factors contribute to their formation	The calcium oxalate stones were found to be associated with systemic diseases (like hypertension, diabetes, and obesity), highlighting the role of dietary and lifestyle changes in their occurrence, recurrence, and possible prevention	Recurrent urinary tract infection occurs due to high rates of stones, such as calcium oxalate, calcium phosphate uric acid frequency	Metabolic profiles (including blood calcium, phosphate, magnesium, creatinine, uric acid, sodium, and potassium) should be measured, and a detailed urinalysis should be done
Alelign et al. 2018, Ethiopia [18]	Expository cohort study (qualitative)	Men and women within the age of 20–49 years	The most common type of kidney stone is calcium oxalate, formed at Randall’s plaque on the renal papillary surfaces	The mechanism of stone formation and recurrence is a complex process which results from several physicochemical events, including supersaturation, nucleation, growth, aggregation, and retention of urinary stone constituents within tubular cells	Due to renal cell injury, plaque is exposed to supersaturated urine. Renal epithelial cell damage (degradation) products promote heterogeneous nucleation and promote crystal adherence in renal cells within 4-6 months	Effective kidney stone recurrence prevention depends upon addressing the cause of stone formation. Generally, to prevent the recurrence of episodes of kidney stone formation or its secondary episodes, proper management of diet and the use of medications is required
D’Costa et al. 2019, USA [19]	Prospective cohort studies (qualitative)	347 patients with asymptomatic kidney stones (mean diameter of 4.4 mm)	The study identified that stone is rather a complex one, which is known as a mobile kidney stone (not yet migrated into the ureter). It may also be symptomatic if it causes intermittent obstruction within the kidney itself	Recurrence of confirmed symptomatic kidney stone episodes resulting in clinical care among first-time symptomatic stone formers was 11%, 20%, 31%, and 39% at 2, 5, 10, and 15 years, respectively	More recently, the five-year symptomatic recurrence rate after the first, second, third, and fourth or higher stone episodes was identified as 17%, 32%, 47%, and 60%, respectively	Risk factors for recurrence were younger age, male gender, white race, family history of stones, gross haematuria, prior asymptomatic stone on imaging, prior suspected stone episode (symptoms but no documented seen stone), a concurrent non-obstructing kidney stone, symptomatic pelvic or lower pole kidney stone, no ureterovesical junction stone, and uric acid stone composition
Fontenelle et al. 2019, Brazil [20]	Expository retrospective cohort study (quantitative)	20 adults from 1994 to 2010	The common types of stones identified in the study include calcium oxalate, calcium phosphate, cystine struvite (magnesium ammonium phosphate), and uric acid. The stones were larger than 10 mm	Recurrence was defined in terms of conservative management, which consists of pain control, medical expulsive therapy with an alpha blocker, and follow-up imaging within 14 days to monitor stone position and assess for hydronephrosis	Asymptomatic kidney stones are recommended to be followed with serial imaging and be removed in case of growth, symptoms, urinary obstruction, recurrent infections, or lack of access to healthcare	Lifestyle modifications such as increased fluid intake should be recommended for all patients, and thiazide diuretics, allopurinol, or citrates should be prescribed for patients with recurrent calcium stones
Nevo et al. 2019, Canada [21]	Retrospective cohort study (qualitative)	Database of 1,328 patients undergoing ureteroscopy and percutaneous nephrolithotomy between 2010 and 2015	Calcium oxalate, uric acid, and struvite stones were found in 298 (65.2%), 99 (21.7%), and 28 (6.1%) patients, respectively	Recurrence was defined in terms of preoperative stone burden was associated with a shorter time to recurrence. During a median follow-up of 38 months (interquartile range [IQR] 31–48), stone recurred in 111 (24%) patients.	One-year stone-free rates stratified by composition were: CaOx 98%, UA 91.9%, calcium phosphate 90%, struvite 88%, and cystine 83%; the two-year stone-free rates were 92.6%, 82.7%, 80%, 73%, and 75%, respectively	UA stone-formers and these other differences are more prominent during the first year of follow-up and should be incorporated into the patient’s follow-up protocol
Lane et al. 2020, UK [22]	Evidence acquisition retrospective cohort study (qualitative)	Studies which recorded operative times of ureteroscopy and stone treatment and correlated them with complications of the procedure	Renal stones were found in 50 minutes (range = 33–75 minutes) compared with 40 minutes (range = 25–60 minutes) for those without a complication	The difference in the complication rates over a total of 15 years [21]. Unsurprisingly, over this time period, operative time reduced from a mean of 75 down to 36.5 minutes, and complication rates reduced synchronously	The single use of ureteroscopes correlated with almost a 15-minute reduction in operative time [19]. The paper did not specify how LithoVue reduced operative time; however, both groups had similar complication rates	The study looked into whether ultrasound-guided URS with holmium laser lithotripsy can achieve the same results as fluoroscopy [15,18]. Thus, the authors recommended that clinicians consider reducing radiation dosage with ureteroscopy
Kim et al. 2023, USA [23]	Retrospective cohort study (qualitative)	Patients with renal or ureteral stones treated with ureteroscopy between 2016 and 2021 from the Michigan Urological Surgery Improvement Collaborative (MUSIC) clinical registry	Ureteral stones were large in proportion, which was about ≥9 mm. The stones identified were ureteral in ureteroscopic lithotripsy	The recurrence definition was considered from evidence that patients who adhere to targeted medical treatment seem to experience less stone growth or regrowth	Calcium oxalate stones, with a high risk of recurrence and complications	The study and data variables were considered inconclusive, as not all patients completed four weeks of treatment
Ripa et al. 2023, UK [24]	Retrospective cohort study (quantitative)	178 patients over a 10-year period	Residual stone fragments were identified in the study, and the median cumulative stone size was 10 mm (7–17.25). The most common locations were the lower pole (18.9%) and proximal ureter (14.9%)	The lack of recurrence was defined as the absence of symptoms and a negative urine culture during follow-up. The study hypothesised that the active treatment has a mean follow-up of 14-3 months.	Among 28 patients presenting with a single symptomatic stone <10 mm in any location, a complete resolution was observed in 85.7% of the cohort. Also, no significant differences in symptom resolution were found between patients with stones	The authors recommended that patients with symptomatic, small renal stones should be offered endoscopic treatment, as the cessation of symptoms was not associated with a specific stone composition.
Achar et al. 2024, India [25]	Cross-sectional study (quantitative)	72 patients having ureteric calculi undergoing URSL	Ureteral stones in ureteroscopic lithotripsy. Ureteral stones were large in proportion, which was about ≥10 mm	The recurrence definition was declared to be based on stone factors such as location, size, composition, presence, and degree of obstruction. The follow-up duration was suggested to be influenced by clinical factors and technical factors. However, an estimated duration of follow-up could be 4 weeks	The study revealed that the recurrence rate of ureteric stones is high, as the stones fail to respond after four weeks of MET	Despite being a small volume, single-centred study, blinding could not be achieved. Therefore, not all patients completed four weeks of treatment
